# Map of Health Needs – basis for the national strategic frameworks

**DOI:** 10.1093/eurpub/ckac131.548

**Published:** 2022-10-25

**Authors:** J Olminski, M Koziol-Rostkowska, A Smiglewska

**Affiliations:** Department of Analyses and Strategies, Ministry of Health, Warsaw, Poland

## Abstract

As the economies transform into data-centred, so do the health systems. Responding effectively to emerging challenges through right decisions and setting up adjusted policies require a well-functioning evidence-based tool. The one developed in Poland is called ‘Maps of Health Needs'. It provides key stakeholders with unbiased data to support decision making processes. The Map depicts the current situation in the system, and forecasts the changes in upcoming years. The system is shown widely: starting with the background of demography and epidemiology, later moving to the out- and in-patient care, including long term care and rehabilitation, concluding with resources (staff and infrastructure). This broad and unbiased view on the system now and in the years to come, allows the public sector institutions to plan the short, medium and long term actions. It regards both strategic and operational levels, for the whole country and for the regions respectively. The documents juxtapose the current and the desired state of the system in the future, outlining necessary actions and goals in order to achieve it. In the recent years a few strategic frameworks have been developed in Poland, as the preparation for the next EU programming period advanced and in reaction to the pandemic crisis. The main documents include:

1) Healthy Future, the umbrella-like framework for the period 2021-2027,

2) Recovery and resilience plan,

3) Transformation plans.

The first and the second one became a part of the negotiation conditions at the European level, as they form a reliable source for modelling the flux of investments’ financing. In that way the maps shape the national policies, becoming the basis for strategic frameworks.

**Key messages:**

• Reliable data is nowadays a key factor in strategic planning, also in the healthcare system.

• The tool developed in Poland, called Maps of Health Needs, serves as a base for strategic national frameworks.

